# Nutrient consumption and chain tuning in diatoms exposed to storm-like turbulence

**DOI:** 10.1038/s41598-017-02084-6

**Published:** 2017-05-12

**Authors:** Gianluca Dell’Aquila, Maria I. Ferrante, Marco Gherardi, Marco Cosentino Lagomarsino, Maurizio Ribera d’Alcalà, Daniele Iudicone, Alberto Amato

**Affiliations:** 1 Stazione Zoologica Anton Dohrn, Department of Integrative Marine Ecology, Villa Comunale, 80121 Naples, Italy; 2 Dipartimento di Fisica, Università di Milano, Via Celoria 16, 20133 Milan, Italy; 3 UMR 7238 CNRS Computational and Quantitative Biology, University Pierre et Marie Curie, 15, rue de l’Ecole de Médecine, 75006 Paris, France; 4 Zellbiologie Philipps-Universität Marburg, Karl-von-Frisch Str., 8 35043 Marburg, Germany; 5 Laboratoire de Physiologie Cellulaire et Végétale, UMR5168 CNRS-CEA-INRA-Université de Grenoble Alpes, Institut de Recherche en Science et Technologies pour le Vivant, CEA Grenoble, 17 rue des Martyrs, 38054 Grenoble Cédex 9, France

## Abstract

Current information on the response of phytoplankton to turbulence is linked to cell size and nutrient availability. Diatoms are considered to be favored by mixing as dissolved nutrients are more easily accessible for non-motile cells. We investigated how diatoms exploit microscale turbulence under nutrient repletion and depletion conditions. Here, we show that the chain-forming diatom *Chaetoceros decipiens*, continues to take up phosphorus and carbon even when silicon is depleted during turbulence. Our findings indicate that upon silica depletion, during turbulence, chain spectra of *C. decipiens* remained unchanged. We show here that longer chains are maintained during turbulence upon silica depletion whereas under still conditions, shorter chains are enriched. We interpret this as a sign of good physiological state leading to a delay of culture senescence. Our results show that *C. decipiens* senses and responds to turbulence both in nutrient repletion and depletion. This response is noteworthy due to the small size of the species. The coupling between turbulence and biological response that we depict here may have significant ecological implications. Considering the predicted increase of storms in Northern latitudes this response might modify community structure and succession. Our results partly corroborate Margalef’s mandala and provide additional explanations for that conceptualization.

## Introduction

Diatoms are unicellular, non-flagellated algae surrounded by a characteristic siliceous exoskeleton called the frustule. This class of algae displays one of the highest species richness in phytoplankton with ca. 14,000 described species^1^ and a growing evidence for the existence of cryptic species^2, 3^. Diatoms’ ecological^4–6^ and, lately, also biotechnological^7–9^ importance is undeniable. Life forms and their concurrent life and cell cycles in diatoms are highly diversified and in several cases rather peculiar^10–12^; at mitosis daughter cells of chain forming species can either separate completely or elongate the chain. Chain formation and the junction mode between two adjacent cells are species-specific features^13–17^ and in some cases, e.g., in many *Chaetoceros* species, the elongation of the chain or its splitting is prepared before cell division by changing the shape of the connecting spines^18–20^, and can be easily monitored looking at that trait.

Diatoms, especially the chain forming ones, are considered to optimally thrive in turbulent environments. The unifying explanation for this is that turbulence may compensate for their lack of self-propelling organs favoring their encounter with dissolved nutrients and their persistence in the euphotic zone^21^. Chain formation as well has been interpreted as a mechanism to heighten the same processes, but a convincing proof and the possible mechanisms behind it, are still elusive (see refs 22–24 for a discussion). It is worth remembering that diatom cell sizes are much smaller than the Kolmogorov scale as well as Batchelor scale in some cases, below which viscosity rules the flow regime. Current knowledge about turbulence response in phytoplankton is strictly correlated to cell size and nutrient availability; e.g. the giant diatom *Coscinodiscus* benefits from turbulence in phosphorus limiting condition compared to the smaller *Thalassiosira pseudonana* ^25^. Barton and coworkers^26^, in a model study concluded that in nutrient replete condition, turbulence does not significantly affect the community structure. Otherwise in low nutrient conditions, turbulence enhances the uptake in larger cells. According to these results, chain length fine-tuning can be a solution to escape the viscosity cage. Chain formation must have an adaptive advantage since thousands of species (not only diatoms) have evolved and retained such a feature. One advantage could be the increase of nutrient uptake, thus reducing the problem of the microzone around cells and chains^27^. For a given level of turbulence, oscillating flow along different parts of a chain will increase nutrient uptake as a function of chain length^27^.

A few *in situ* evidences of the link between turbulence, i.e. mixing, and phytoplankton community compositions, especially during blooms, exist^28, 29^. For historical and ecological reasons^30^ a significant amount of information on diatom and phytoplankton blooms come from the North Atlantic (NA). Comparing the pre-blooming physical conditions and biological assemblages in two different areas of that basin, several authors noted that where turbulence intermittently acted before the bloom, mini-blooms mainly composed of diatoms dominated by *Chaetoceros* spp, occurred and that after the mini-bloom demise the community structure remained dominated by diatoms. In the area where no turbulence pulses occurred, the situation was completely different^31^.

The aim of the present work is to show how the diatom *Chaetoceros decipiens* responds to turbulence both in terms of nutrient uptake and chain formation. We chose this species because it belongs to the most recurrent diatom genus in the world ocean^32^ and because in this species chain splitting is morphologically identifiable by the presence of a so-called separation point and therefore can be used to follow chain formation dynamics during growth. A separation point in a chain is constituted by two adjacent cells that have synthesized cellular processes morphologically different from those synthesized by the other cells in the chain^20^.

Experiments have been carried out in nutrient repletion and lasted until nutrients were depleted. This has enabled us to follow how microscale turbulence influenced the switch from one condition to the other.

## Results

### Growth, pH and nutrient consumption

**Figure 1:**
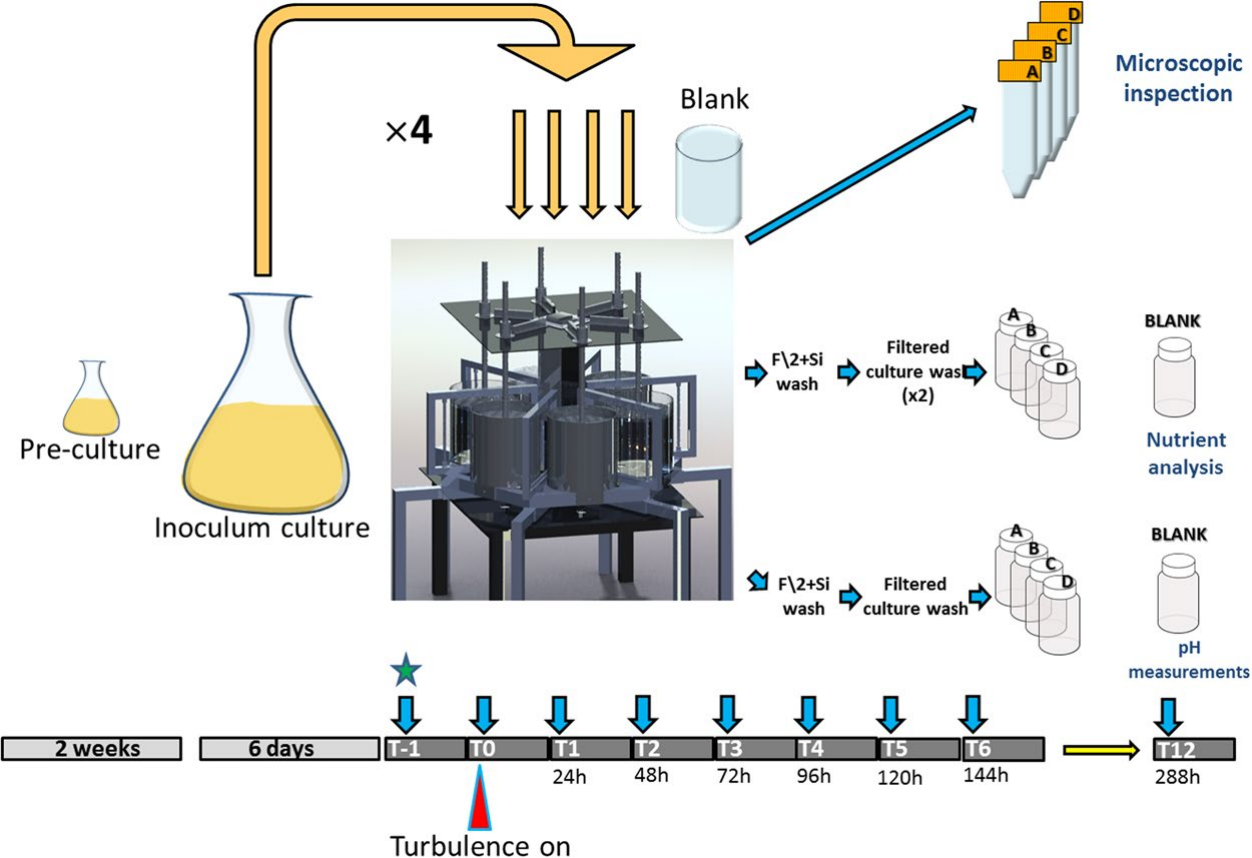
Graphic representation of experimental setup. Note: at T-1 (green star) samples were used for pH measurements only.

**Figure 2:**
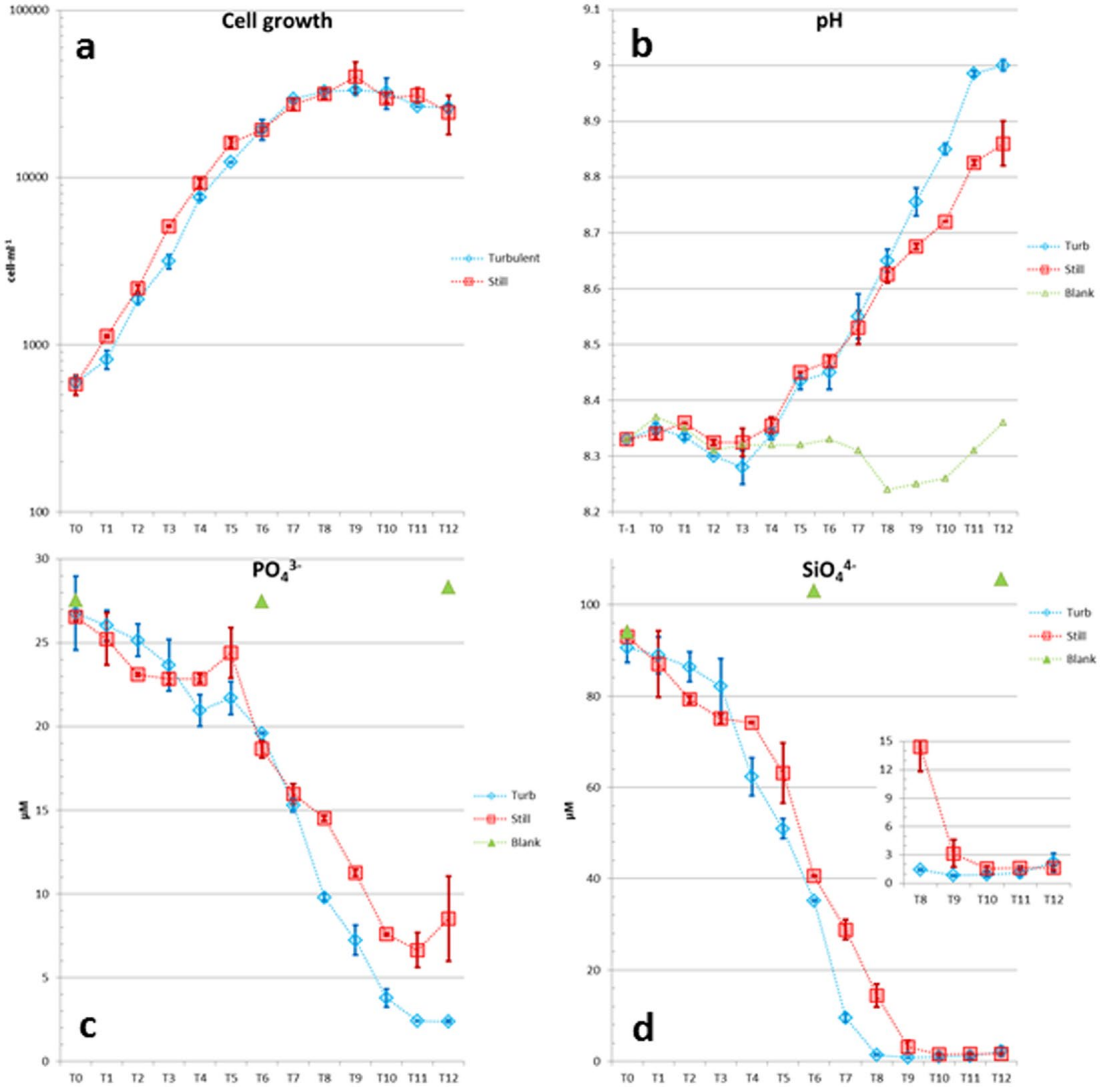
Growth curves (**a**), pH values (**b**) and nutrient concentrations (**c, d**) over the experimental days (T0-T12). (**a**). Cell concentrations over the experimental days. Logarithmic y axis. (**b**). Mean pH measurements. Still cylinders were always gently stirred before sampling (see methods). (**c**). Phosphate and (**d**). silicate measurements in Turbulent and Still conditions. Inset: zoom-in of the portion between T8 and T12. In the turbulent condition the threshold silicate concentration (ca. 1–2 µM^33, 34^) for diatoms was reached. Abscissa always represents experimental days. Values are means of the duplicates (n = 2) and vertical bars indicate maximum and minimum values. Blue diamonds indicate turbulent condition, red squares the still condition and, where applicable, triangles indicate measurements in the blank (supernumerary beaker where no cells were inoculated, see Methods).

The *Chaetoceros decipiens* strain SZN-Cdec was grown in quadruplicate cultures, two exposed to microscale turbulence and two in still condition for 13 days (Fig. 1). Both turbulence-exposed and control cultures presented an exponential growth phase lasting until T6 and a clear change in the slope at T7 (Fig. 2a), which we may consider as the end of the exponential phase. The two sets of duplicates show little, statistically insignificant (accuracy 99.9%), differences in cell growth (Supplementary Table 1), as also demonstrated from the division rates calculated on the three portions of the growth curve (exponential, stationary and post-exponential phases, Table 1).

**Table 1:** Division rates expressed as divisions·day^−1^ calculated in the three portions of the growth curves, i.e. the exponential (T1–T6), stationary (T7–T9) and post-exponential (T10–T12) phases.

Experimental condition	Turbulent		Still	
Period (days)/cylinder	A	B	C	D
T1-T6	0.90	0.88	0.89	0.90
T7-T9	0.10	0.48	0.07	0.05
T10-T12	0.05	−0.02	−0.34	−0.31

An asterisk indicate that replicates are not statistically different (accuracy 99.9%).

Daily division rates, expressed as cell divisions per day (Supplementary Table 1), showed an increase at the very beginning of the experiment with maxima recorded at T4 in turbulence-exposed and T3 in control condition. Afterwards, the division rates decreased steadily until T7. The overall division rate, calculated on the steepest portions of the growth curves were 0.99 and 0.98 divisions per day in turbulent and still condition, respectively.

pH values were recorded in the medium of the four cell suspensions along the experiment to infer carbon fixation. From T0 to T8, the pH curves of turbulence-treated and control conditions overlapped (Fig. 2b). From T9 pH in turbulence-exposed cultures rose faster than control duplicates until the end of the experiment (Supplementary Tables 2, 3), hence we hypothesize a stronger carbon fixation in turbulence. Blank did not show any significant variation (Supplementary Table 4) with a mean pH value of 8.31+/− 0.039 (mean +/− SD).

Nitrate (NO_3_^−^), phosphate (PO_4_^3−^) and silicate (SiO_4_^4−^) concentrations were measured in turbulent and still duplicates. NO_3_^−^ concentration was always above 700 µM and the effect size measure (Cohen’s *d*) showed that turbulence had no effect on it. This possibly revealed no variation to nitrate uptake driven by turbulence. Average NO_3_^−^ concentrations were never statistically different between turbulence-exposed and control cultures (*p*-value 0.20, accuracy 95%). Phosphate (PO_4_^3−^) steadily diminished over the experimental days in both turbulence-exposed and control cultures (Fig. 2c). At T8 the phosphate consumption diverged between still and turbulent conditions, with turbulence-exposed cultures taking up more phosphate than the control (*p*-value 0.02, 95% accuracy). At the end of the experiment, while cell concentration between treated and control samples were comparable (2.6·10^4^ and 2.5·10^4^ cells·ml^−1^ in the turbulent and still conditions, respectively), PO_4_^3−^ concentration differed by a factor >3, revealing a more intense phosphate consumption in turbulent-exposed cells (Cohen’s *d* 1.44 over the period T8–T12 reveals a very strong effect size). Silicate was the only nutrient among those measured that reached concentrations considered to be limiting for diatoms (1–2 µM^33, 34^). This occurred at T9 in control and at T8 in turbulence-exposed cultures (Fig. 2d). Silicate concentration and uptake, were highly affected by turbulence (*p*-value 0.02, 95% accuracy, Cohen’s *d* 1.87).

In order to mathematically compare the effect of turbulence on nutrient consumption, nutrient concentrations over time were fitted with the following phenomenological sigmoidal function using the non-linear least squares regression:

1 $$[{\rm{Nutrient}}]\cong A\cdot {e}^{-{(t/{t}_{0})}^{\gamma }}$$

where [Nutrient] is the nutrient concentration (Si or P), *A* is the initial nutrient concentration, *t* is the time, *t*_*0*_ is a characteristic decay time, indicating when the nutrient concentration reaches the value *e* ^−1^ and *γ* is a parameter that characterizes the increase with time of the absorption rate. *γ* reflects the increase in cell number with time. Correlation coefficient (r), root-mean-square relative error (RMSE) and Theil’s U coefficient were calculated for all the fits (Table 2). Equation (1) can be seen as resulting from a non-homogeneous Poisson (absorption) process with power-law increasing rate.

**Table 2:** Correlation coefficient (r), RMS relative error (RMSE), Theil’s U coefficient for the non-linear least squares regression fits performed on P and Si concentrations during the experiment.

Nutrient	Condition	r	RMSE	Theil’s U
Phosphate	Still	0.96	0.15	0.10
	Turbulent	0.99	0.18	0.08
Silicate	Still	0.99	0.53	0.08
	Turbulent	0.99	0.77	0.07

Remarkably γ = 3.8 for all fits. Conversely, the fitted characteristic decay times *t*_*0*_ differed for both silicate and phosphate between still and turbulent conditions, namely *t*_*0 Si still*_ = 6.7 while *t*_*0 Si Turbulent*_ = 5.8 and *t*_*0 P still*_ = 10.1 while *t*_*0 P turbulent*_ = 8.4.

### Chain spectra

**Figure 3:**
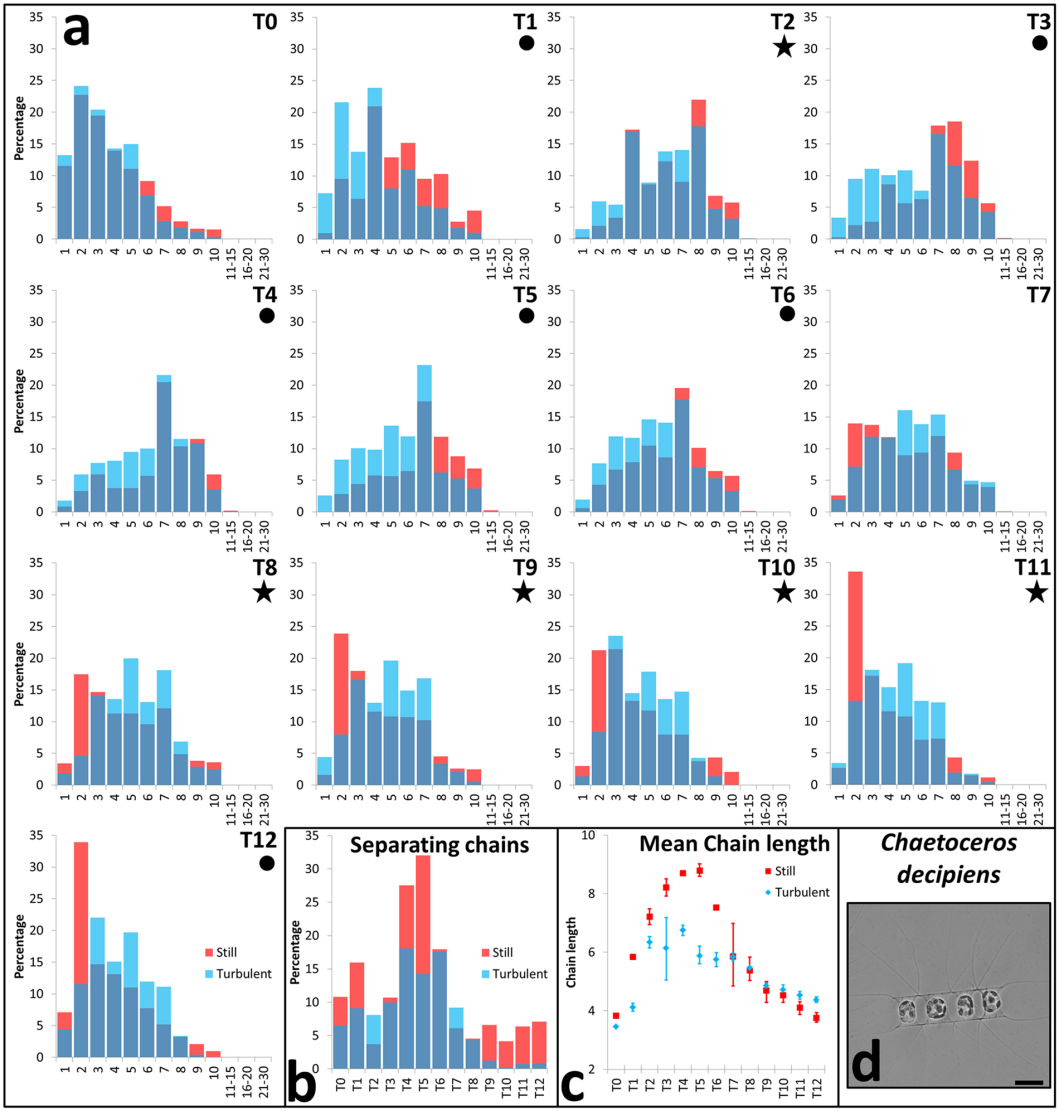
Chain spectra over experimental days (T0–T12) (**a**), separating chain frequency (**b**), mean chain length (**c**), in the centric diatom *C. decipiens* (**d**). (**a**) Abscissa represents the chain length expressed as number of cells per chain, ordinate the frequency of each chain class expressed in percentage. All results were statistically validated by Kolmogorov-Smirnov two-sample (KS2) and Wilcoxon non parametric tests (Supplementary Table 5). A full circle indicate that both test responses equal 1 with a *p*-value <10^−10^, a black star indicate *p*-value between 10^−4^ and 10^−10^, no symbols below the time point indicate that both test responses equal 0 with a *p*-value above 10^−3^. KS2 and Wilcoxon tests were also run in order to test statistical robustness of biological replicates. Tests were run in pairwise combinations between replicates (Supplementary Table 5). Dark blue portions of the histograms indicate overlap, red histograms indicate enrichment of a given chain class in the still condition, blue histograms indicate enrichment in the turbulent condition. (**b**) Abscissa represents the time points expressed in days (T0–T12). (**c**) Mean chain length over time (Abscissa represents time points). Vertical bars indicate maximum and minimum values (n = 2). (**d**) a *Chaetoceros decipiens* 4-celled chain. Scale bar indicate 20 µm.

Chain length dynamics and separating chain frequency were followed in control and turbulent cultures. At T0, chain spectra (Fig. 3a) in turbulent and still conditions overlapped (verified by statistical tests, Supplementary Table 5). From T1 to T5 short chains in turbulence became enriched more than in still conditions. This finding is corroborated by the separating chain frequency (Fig. 3b), i.e. the percentage of chains presenting at least one separation point (see introduction), and by mean chain length (Fig. 3c). From T7 until the end, turbulence-exposed cultures showed almost stable chain composition with mean chain length decreasing more slowly than in control cultures (Fig. 3c). In the latter, chain spectra tended to be enriched in shorter chains (Fig. 3a).

**Figure 4:**
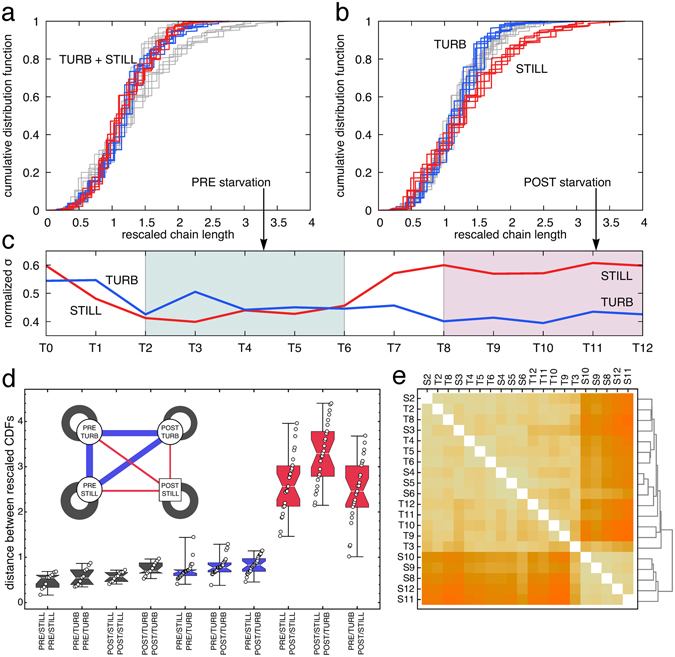
Comparison of chain-length distributions. (**a, b**) Cumulative distribution functions (CDF) rescaled by mean chain length before (**a**) and after (**b**) starvation; turbulent conditions are in blue, still conditions in red. The grey lines in a. are the colored ones in (**b**) and vice-versa. (**c**) The dynamics of the normalized standard deviation for the two conditions throughout the experimental time points (from T0 to T12 on the x-axis). Green and pink colors indicate PRE- and POST-starvation groups used in the analyses. (**d**) The L^2^ distance (integral of squared differences) between the cumulative distribution functions for all pairs of CDFs (circles). The four different combinations, PRE- and POST-starvation in still and turbulent conditions (corresponding to the four nodes in the inset), are contrasted pairwise, yielding ten possible combinations (corresponding to the groupings on the x-axis and to the links in the inset, whose width is inversely proportional to the average distance). Intra-combination distances (dark grey in figure) are smaller than inter-combination distances involving POST-still (red in figure), but comparable to the other inter-combination distances (blue in Figure). (**e**) Hierarchical clustering based on the L^2^ distances (darker shades correspond to larger distances) isolates the four POST-starvation CDFs in still conditions (S8–S12). The other cluster groups S2–S8 (pre-starvation in still) and all the turbulent CDFs (T2–T12). Clustering does not discern the POST-starvation CDFs in turbulent conditions (T8–T12).

In order to further explore the different behaviours in terms of chain spectra, data were compared by dividing them into two sets: from T2 to T6 (named PRE-starvation) and from T8 to T12 (POST-starvation). Chain lengths were rescaled by mean length (separately for each spectrum). The cumulative distribution functions of such rescaled chain spectra were compared for turbulent and still conditions both PRE- and POST-starvation (four combinations in total). In the period of nutrient repletion, distributions from still and turbulent cultures all collapsed on the same curve (Fig. 4a), while after nutrient depletion the still and turbulent cultures collapsed on two different curves (Fig. 4b). Similarly, the normalized standard deviations of the chain lengths (Fig. 4c) were divergent for the two cultures after nutrient depletion (note that the opposite happened for the mean, Fig. 3c). This is validated by the Euclidean distance (L^2^ distance) calculated in pairwise comparisons between the four combinations mentioned before (Fig. 4d). Taking the variance of the internal distances (e.g. PRE-still vs PRE-still) as a reference background noise, 98.7% of the heterogeneous distances involving POST-still (e.g. POST-still vs POST-turbo) were off by more than 3σ, as opposed to 6.7% of the other distances (e.g. PRE-still vs POST-turbo). From this analysis, corroborated by a hierarchical cluster analysis (Fig. 4e), it is clear that upon nutrient limitation cultures showed a divergent behavior, with the turbulent condition (POST-turbo) sustaining chain spectra similar to the nutrient-replete period.

## Discussion

**Figure 5:**
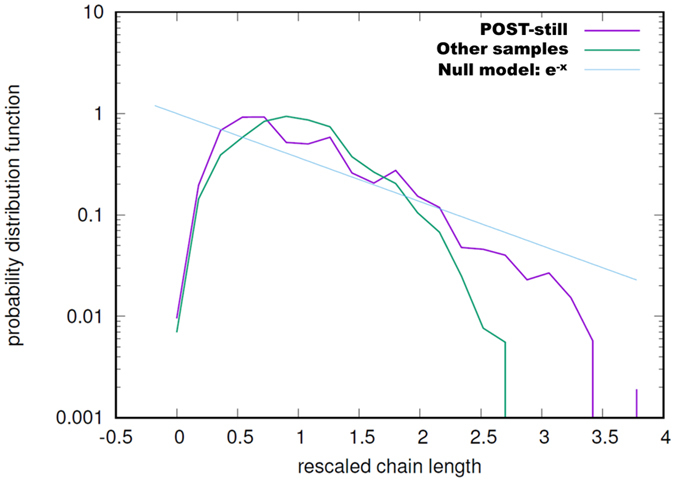
Chain spectra in POST-still cluster (see Fig. 4) and in all the other clusters taken as a single group compared to the null model in which chain separation is stochastically regulated^42^.

Our results of nutrient concentration, pH measurements (Fig. 2), and chain spectra (Figs 3–5), show that, when suspended in a turbulent fluid, *Chaetoceros decipiens* cells behave differently than in a still fluid. In turbulence, nutrients are taken up more vigorously and upon silica depletion, carbon is more strongly fixed. The physiological state of the cells (inferred by chain length distribution) as well appears enhanced in turbulence. Such a response to turbulence is remarkable, considering the small size of the species (valve diameter size ranging 23–25 µm), well below the Kolmogorov length and in the same order of magnitude of the Batchelor scale. Compared to previous investigations (e.g. refs 25, 35 and 36) our results show how microscale turbulence influences cell response to a switch from nutrient repletion to nutrient limitation. The effect of turbulence on organisms below the Kolmogorov scale is still an open question and Peters & Marrasé^37^ suggest that this effect is produced by residual laminar shear field. In our experiments, the shear rate calculated following Karp-Boss and co-workers^22^ is ca. 0.22 s^−1^. This shear rate was demonstrated not to affect N uptake in the dinoflagellate *Karenia brevis* ^38^ and we assume it not to be perceived by *C. decipiens* either. In essence, our experiments demonstrate that *C. decipiens* directly perceived turbulence both in nutrient repletion (like e.g. *Thalassiosira rotula* ^39^) and in starvation and responded to it.

In nutrient replete conditions the differences are less pronounced. A weak sign could be the time lag in reaching a similar division rate on days 1–2 (Supplementary Table 1) as reported for the small non-colonial diatom *Thalassiosira pseudonana* in P-limited conditions^25^. Although the conditions are different and the species investigated was much smaller (ca. 6 µm in diameter) than *C. decipiens*, a weak lag phase in turbulence-exposed cultures was identified in that series of experiments as well without any explanation^25^. In *C. decipiens*, this lag phase of growth might be explained in the light of previous results^39^. A similar experiment was carried out with the same strain and RNA-seq was performed. A differential expression analysis, validated by qPCR, showed that at the beginning of turbulence exposure (namely at T2), genes involved in energy storage were up-regulated in turbulence and this was interpreted as storage of exceeding energy^39^. In fact, the regulation pattern displayed by *C. decipiens* in turbulence was similar to that recorded in *Phaeodactylum tricornutum* at the onset of light phase, i.e. when light energy reactivates different metabolisms^40^.

More significant are the faster uptake rates, e.g., the *t*_*0*_ and *γ* values (Eq. 1), and the chain length regulation (Fig. 3). Through a mathematical approach to our empirical data, it was possible to corroborate our results. In Eq. 1, the fitted characteristic decay time *t*_*0*_ diverged for silicate and phosphate in still vs turbulent conditions, while *γ* did not. This suggests that all the cultures were following the same rule in the uptake rate in relation to their increase in cell number (equal *γ*) but in turbulence, *C. decipiens* cells took up Si and P faster (different *t*_*0*_). The fit assumes that the uptake rate changes with time with a tunable power law *t* ^*1*/*γ*^. The fact that the parameter *γ* is nearly the same for all fits, supports the hypothesis that the process that governs the change in the uptake with time (likely due to the decrease of available nutrients) is the same in different conditions. However, the characteristic time *t*_*0*_ (the half-life time) of nutrients changes with turbulent vs still conditions indicating a clear effect of this treatment on the ability to take up nutrients.

When silicate ran out a marked change in the chain spectrum took place (Fig. 3), with a tendency to produce shorter chains in still, which we interpret as a sign of senescence^41^. By contrast, in turbulence cells produced significantly longer chains, which we interpret as a good physiological state^41^. It has been mathematically demonstrated that *C. decipiens* exerts an active control on chain length dynamics during growth^42^. Chain length spectrum from exponentially growing *C*. *decipiens* was compared with that obtained from the null hypothesis of the model, i.e. a spectrum produced by a cell culture where chain elongation and separation occur stochastically. Comparing the chain spectra from turbulent and still conditions with that generated by the null-model, it emerged that POST-still samples are closer to null-model than all the others (Fig. 5). We interpret this as a loss of the tight control on chain regulation. The POST-turbo chain spectra collapse on the same curve as the PRE samples thus we can hypothesize that even upon Si depletion, turbulence-exposed cultures behave like before silicate ran out. The better physiological condition in turbulence-exposed cultures is linked neither to light availability nor to overcrowding because light was homogeneous in all the TURBOGEN cylinders and cell concentrations were comparable, therefore differential self-shading or crowding between treated and control cultures can be ruled out. Although the *C. decipiens* culture was not axenic, we did not monitor bacteria throughout the experiment. The presence of bacteria and their effect can be considered negligible in terms of the differential nutrient consumption that we scored in turbulence vs still duplicates. It has been demonstrated that for small cells, the Sherwood number stays ca. 1 over a wide range of ε, namely from 10^−4^ to 10^−10^ m^2^s^−3^ ^22^. P and N uptake in bacteria varies of ca. 0.6%^22^ and 0.2%^43^ respectively, in presence or absence of turbulence. Microscale turbulence is the only factor the difference in nutrient consumption and chain spectra can be ascribed to.

In turbulence, *C. decipiens* metabolism is accelerated even during the early exposure to the mechanical stimulus^39^ and, in general, it appears to boost the cell machinery for nutrient uptake and carbon assimilation. The latter is inferred from pH variations. Upon silicate depletion below the tolerance concentration (1–2 µM^33, 34^ for diatoms), carbon was fixed more vigorously in turbulence and P taken up faster. The absence of silicate in the medium impeded active cell division (Fig. 2a) but did not hamper carbon sequestration (Fig. 2b) and phosphate uptake (Fig. 2c,d). Carbon fixation and growth are independent phenomena^25^ and cells ‘can keep fixing C beyond the possibility to divide’^25^. In this condition, turbulence-exposed cultures fix more C and take up more P than controls. In still, the flux of nutrients is slower whereas in turbulence, cells need more P to balance the internal elemental ratio^25^. The C fixed and the nutrients taken up were presumably used in storage compound production to be eventually converted to energy via the citric acid cycle or to polymer secretion. This, might require further P uptake, even in the absence of cell growth.

Besides the impact on *C. decipiens* in itself, and likely on other diatoms displaying similar biology, our results may impact community dynamics as well. A linear extrapolation of our results to the field must be taken with caution, though. *Chaetoceros* is the most abundant diatom genus in the world ocean^32^ and the capability of our model species to benefit from turbulence, keeping an apparently healthy state even in presence of silicate depletion and being able to remove other nutrients, might give it a competitive advantage on other species or groups. Our results led to the conclusion that microscale turbulence can shape phytoplankton community structure, however, not necessarily in a size-dependent manner^26^. In our investigations (present work and ref. 39) we have shown how different species react differently to microscale turbulence and how this can influence nutrient uptake. Using the equation proposed by MacKenzie & Legget^44^, we estimated that the wind speed associated to the ε imposed in the present work corresponded to ca. 2.6 m·s^−1^, which corresponds to a light breeze. If turbulence levels increase, then diatom prevalence in blooms and in preconditioning, via seeding, future blooms might increase too. Based on remote sensing data, it has been demonstrated that from 1995 cyclonic storm frequency and intensity trends in the NA have shown statistically significant increase^45^. The NA hosts the most important phytoplankton bloom on Earth^30^ and an increase in storms, with an expected increase in turbulence intensity and duration, might be producing a shift in the phytoplankton community. Furthermore, the relative abundance of diatoms with respect to dinoflagellates has increased in the last decade. This phenomenon has been correlated to increased surface water temperature and more windy conditions in summer^46^. As a consequence, biogeochemical cycles of different elements could be affected in the area with turbulence playing an overlooked role in it.

The hypothesis that phytoplankton species succession might depend on the nutrient utilization by preceding species, thus causing an alternation of different groups in a given area, has been recurrently discussed in the literature (e.g. ref. 47). A further support to this scenario could be the evidence of different nutrient affinities or uptake efficiencies by different species^34, 48–50^. In all reconstructions, turbulence would either mitigate the issue of nutrient diffusion to the cell^22–24, 36, 37, 51–55^ or would solve the problem of bulk nutrient flux^20^. Margalef^21, 56, 57^ as well interpreted phytoplankton succession in terms of the seasonal cycle of environmental forcing, phytoplankton life strategies and their biological traits. His observation was that some species cope better with turbulence and his interpretation was that this could be preferentially linked to a counteraction of the sinking rate and improvement of nutrient uptake via optimization of the surface-to-volume-ratios. Our results suggest that *C. decipiens*, which benefits from turbulence in Margalef’s view, thrives very well in turbulence apparently beyond, or not specifically relying on, those mechanisms. In our experiment silica depletion arrested cell division (Fig. 2a–c) without reducing P probably nor N uptake. This, besides suggesting an active physiological state, would also limit the availability of nutrients to other species possibly affecting pelagic ecosystem dynamics^31^. Among the numerous attempts carried out to match *in situ* spring bloom observations with mathematical models^58–60^, mixing has generally been considered as a physical forcing which phytoplankton are passively subjected to and not as a specific benefit for them. Recently, in a model study it has been demonstrated that turbulence does not affect community structure in nutrient repletion conditions while in nutrient limitation, turbulence would enhance the top-down control exerted by predators and balance the physiological disadvantage of larger cells^26^. In our experiments, no predators were present and significant differences in chain spectra (interpreted as a proxy for physiological state of the culture^41^) were recorded both in nutrient repletion and depletion. This corroborates the idea that fluid motion at microscale could be directly perceived by the cells so to become a source of information to eventually trigger and organize the observed physiological responses^61^.

## Methods

### Medium preparation and culture maintenance

The non-axenic *Chaetoceros decipiens* SZN-Cdec culture was established by single chain isolation from net samples collected at the long-term monitoring station MareChiara (LTMS MC^62^) and maintained at 18 ± 1 °C, photoperiod at 12 L:12D and irradiance at 80 μmol photons·m^−2^·s^−1^. Bacteria were not monitored during our experiments because their presence and effects on nutrient consumption was considered to be negligible in turbulent vs still condition. Qualitatively, bacterial load in the cultures was very low until the end of the experiment.

The culture was maintained in F/2 medium^63^, prepared by adding adequate quantities of nutrients from single stock solutions to ultrafiltered (0.22 µm nitrocellulose filter GSWP09000 Merck Millipore Darmstadt, Germany) and sterilized superficial seawater collected at the LTMS MC. For the experiments, the medium was filtered through 0.45 µm (Millipore, HAWP09000) to get rid of the flocculates produced by F/2. The medium was stored in the growth chamber where the experiment was run 48 hours before the start of the experiment.

### Experimental set-up and sampling

The experiment was performed using TURBOGEN^64^, a prototypic instrument that consists of six 13 L Plexiglass^®^ cylinders for algal growth, integrated with a fully digitally controlled high-performance engine that allows vertical movement of circular grids to generate turbulence in the cylinders.

Figure 1 summarizes the experimental plan. The experiment was carried out in duplicate (in accordance with previous studies e.g. ref. 25), two replicates for each treatment for a total of four cylinders used. The SZN-Cdec culture was maintained in exponential growth phase by serial dilutions for two weeks prior the experiment in a 25 cm^3^ TPP^®^ filter screw cap flask (Techno Plastic Products, Trasadingen, Switzerland; cat. n. 90026). One week before the experiment, a 5 L Pyrex^®^ sterile Erlenmeyer flask was inoculated and the growth was monitored for six days. The day of inoculum (T-1), a cell count in the 5 L Erlenmeyer was carried out by Sedgwick-Rafter counting chamber^65^ in a light microscope (Zeiss Axioscope 2 Plus, Carl Zeiss Microscopy, LLC One Zeiss Drive Thornwood, NY 10594 United States) and the cell concentration estimated at 3 × 10^4^ cells·ml^−1^. The total amount of F/2 medium prepared for the experiment (13 L per cylinder × 4 = 52 L) was inoculated with 1.3 × 10^7^ cells to get to a final concentration of 250 cells·ml^−1^. The bulk culture was equally distributed over four cylinders (13 L per cylinder) of the TURBOGEN. Cells were left 24 hours acclimate to the dilution. Temperature, light intensity, and photoperiod used in the experiment were set as the growth chamber used for maintenance. A plastic 5 L beaker was added in the growth chamber and filled with F/2 medium only, to be used as a blank for nutrient concentrations and pH values. Before inoculum, a sample for nutrient and pH measurements was taken following the procedure described below. At time point T0 the four cylinders were gently stirred using a strippette for 15 seconds (~60 r.p.m.) and samples were collected as described below. Turbulence was activated in two cylinders (named A and C) with the following settings: stroke 240 mm, grid speed 100 mm·s^−1^, acceleration 1000 mm·s^−2^, producing a kinetic energy dissipation rate in the order of 10^−5^ m^2^·s^−3^ that is comparable to natural turbulence levels^66^. In the other two cylinders (B and D) grids were not inserted and thus turbulence was not applied in order to have a duplicate negative control (that will be referred to as ‘still conditions’). The residual turbulence due to evaporation-driven convective movements was considered negligible, in line with previous studies^25^.

Every day until time point T12, at 10.00, 10 ml of culture from each cylinder were collected in 15 ml Corning^®^ Falcon tubes (Corning^®^, cat. n. 430790, Corning Incorporated NY 14831, USA) and fixed with neutralized formaldehyde (final concentration 1% v/v) then stored at 4 °C in the dark until microscopic inspection. In order to reduce cell settling, the still cylinders (B and D) were stirred as described above every day. We are confident that this procedure did not alter the results. Stirring lasted 15 s (less than 0.02% of the time) and it has been demonstrated that up to two 5-minute-turbulence pulses per day have no effect on growth^67^. In a pilot experiment, cell counts were carried out in still conditions before and after stirring for the first three days demonstrating that sinking is not relevant^39^. For both pH and nutrient measurements, polyethylene vials were washed once with one volume of sterile F/2 medium and once (twice for nutrient measurements) with one volume of filtered culture using TPP 0.22 µm syringe-filter. For pH measurements, 5 ml of filtered culture were collected in the vials pre-conditioned as described above and analyzed straight away; for nutrient measurements, 15 ml were stored at −20 °C until analysis. pH was measured using a benchtop pH/mV Meter, CyberScan ph-510 (EC-PH510/11S, Thermo Scientific Eutech Inc., Waltham, Massachusetts, USA). Measurements were carried out for all cylinders and for the blank beaker, for all time points.

### Nutrient measurements via Segmented Flow Analysis (SFA)

SFA method was carried out with a microflow automated continuous flow analyser (CFA, Flowsys, Systea S.p.A. Analytical Technologies - Anagni, Italy) using the classical colorimetric methods for nutrient analyses^68, 69^.

### Data analysis

Cell counts were carried out using a Zeiss Axioscope 2 Plus at magnification 125 × (objective 10×; Optovar 1.25×). On average, 400 chains per sample were examined using Sedgwick-Rafter chambers^65^ until time point T6, 263 for the rest (17505 total chains counted over a total of 52 samples analyzed). The number of cells composing each chain was recorded. For chain distribution, every chain length class, expressed as cells per chain, was expressed in percentage (weighted on the total number of chains in the sample). The number of cells was estimated by multiplying the number of chains of a specific size class, by the chain length expressed as cells per chain to obtain the total number of cells counted. Statistics of the Sedgwick-Rafter chambers^65^ were applied to obtain cell concentrations expressed as cell·ml^−1^. The cell counts were used to draw growth curves. The division rate expressed as number of cell divisions per day (day^−1^) was calculated as the slope of the growth curve built with log_2_-transformed cell density values between ensuing time points and on the three different portions of the growth curve, i.e. T1–T6, T7–T9, T10–T12. Maximum division rate was calculated on the steepest portion of the curve, i.e. between T1 and T5.

### Statistical tests

Statistical treatment of the data was performed to validate the results using MatLab^®^ and Mathematica^®^. Kolmogorov-Smirnov and Wilcoxon non parametric tests were performed (accuracy 99.9%, α = 0.001) on chain spectra. All the tests were carried out between replicates (turbulent vs turbulent or still vs still) to confirm their robustness and between controls and treated samples (turbulent vs still). All the possible pairwise comparisons were performed.

## Electronic supplementary material

Supplementary info
