# Supplementary material for: Nutrient consumption and chain tuning in diatoms exposed to storm-like turbulence
Source: Sci Rep. 2017 May 12;7:1828. doi: 10.1038/s41598-017-02084-6 (PMC5431809; doi:10.1038/s41598-017-02084-6)
Supplement: Supplementary file 1 — Supplementary info [file 41598_2017_2084_MOESM1_ESM.doc]

**Nutrient consumption and chain tuning in diatoms exposed to storm-like turbulence**

Gianluca Dell’Aquila, Maria I. Ferrante, Marco Gherardi, Marco Cosentino Lagomarsino, Maurizio Ribera d’Alcalà, Daniele Iudicone, Alberto Amato

**Supplementary Table 1.** Division rates calculated over successive time points in single replicates and by averaging cell counts from duplicates. An asterisk indicate that the samples are not statistically different (accuracy 99.9%).

| **Time point** | **Turbulent*** | | **Still*** | | **Cumulative*** | |
| --- | --- | --- | --- | --- | --- | --- |
|  | **A** | **C** | **B** | **D** | **Turbulent (A-C)** | **Still (B-D)** |
| **T1** | 0.55 | 0.35 | 0.74 | 1.19 | 0.46 | 0.96 |
| **T2** | 0.93 | 1.50 | 0.91 | 0.99 | 1.19 | 0.95 |
| **T3** | 0.98 | 0.52 | 1.30 | 1.18 | 0.75 | 1.24 |
| **T4** | 1.20 | 1.36 | 0.78 | 0.92 | 1.28 | 0.85 |
| **T5** | 0.63 | 0.75 | 0.98 | 0.63 | 0.69 | 0.81 |
| **T6** | 0.85 | 0.44 | 0.21 | 0.30 | 0.66 | 0.26 |
| **T7** | 0.42 | 0.80 | 0.34 | 0.65 | 0.60 | 0.50 |
| **T8** | 0.14 | 0.17 | 0.24 | 0.20 | 0.15 | 0.21 |
| **T9** | 0.06 | -0.03 | 0.72 | -0.10 | 0.02 | 0.34 |
| **T10** | -0.41 | 0.29 | -0.62 | -0.17 | -0.03 | -0.43 |
| **T11** | 0.05 | -0.57 | 0.10 | 2.59·10^-3^ | -0.29 | 0.06 |
| **T12** | 0.05 | -0.10 | -0.14 | -0.62 | -0.03 | -0.34 |

**Supplementary Table 2. pH values reported for turbulent (A, C), still (D and F) and in the blank cylinders.** Mean and σ are reported. Underlined are the values used for Student's t-test. The mean values and σ calculated over T9-T12 (last two rows) were used for effect size measure (Cohen's *d*) reported in Supplementary Table 3.

| **Time point** | **Cylinder** | | | | |
| --- | --- | --- | --- | --- | --- |
|  | **A** | **B** | **C** | **D** | **Blank** |
| **T-1** | 8.33 | 8.33 | 8.33 | 8.33 | 8.33 |
| **T0** | 8.36 | 8.35 | 8.34 | 8.33 | 8.37 |
| **T1** | 8.34 | 8.36 | 8.33 | 8.36 | 8.35 |
| **T2** | 8.30 | 8.32 | 8.30 | 8.33 | 8.31 |
| **T3** | 8.31 | 8.35 | 8.25 | 8.30 | 8.32 |
| **T4** | 8.33 | 8.34 | 8.35 | 8.37 | 8.32 |
| **T5** | 8.45 | 8.45 | 8.42 | 8.45 | 8.32 |
| **T6** | 8.42 | 8.46 | 8.48 | 8.48 | 8.33 |
| **T7** | 8.59 | 8.50 | 8.51 | 8.56 | 8.31 |
| **T8** | 8.67 | 8.64 | 8.63 | 8.61 | 8.24 |
| **T9** | 8.73 | 8.68 | 8.78 | 8.67 | 8.25 |
| **T10** | 8.84 | 8.72 | 8.86 | 8.72 | 8.26 |
| **T11** | 8.98 | 8.83 | 8.99 | 8.82 | 8.31 |
| **T12** | 8.99 | 8.90 | 9.01 | 8.82 | 8.36 |
| **Mean** | **8.56** | **8.52** | **8.54** | **8.51** | **8.31** |
| **σ** | **0.253** | **0.200** | **0.266** | **0.189** | **0.039** |
| Mean (T9-T12) | 8.89 | 8.78 | 8.91 | 8.76 | **-** |
| σ (T9-T12) | 0.124 | 0.101 | 0.109 | 0.075 | **-** |

**Supplementary Table 3. Student's *t*-test carried out on pH values from time point T9 to T12 (i.e. when the two pH curves diverge, Fig. 1b), accuracy 90%.** Cohen's d values reflect the appropriate effect size measure if σ of the two samples are comparable. Red digits indicate that the two samples analyzed are not significantly different. In bold-face the internal controls are reported, i.e. statistical tests carried out between replicates (turbulent cylinder A vs. turbulent cylinder C and still cylinder B vs. still cylinder D) in order to validate robustness of replicates.

| **Samples** | ***t*-test outcome** | ***p*-value** | **Cohen's d** |
| --- | --- | --- | --- |
| **A vs B** | 1.28 | 0.1234 | 0.91 |
| **A vs D** | 1.76 | 6.44×10^-2^ | 1.24 |
| **C vs B** | 1.72 | 6.85×10^-2^ | 1.21 |
| **C vs D** | 2.30 | 3.05×10^-2^ | 1.63 |
| **Internal controls** | | | |
| **A vs C** | **-0.30** | **0.3862** | **0.21** |
| **B vs D** | **0.40** | **0.3522** | **0.28** |

**Supplementary Table 4. Student's *t*-test carried out on pH values, accuracy 99.9%.** Glass' Δ values reflect the appropriate effect size measure if σ of the two samples are not comparable.

| **Samples** | ***t*-test outcome** | ***p*-value** | **Glass' Δ** |
| --- | --- | --- | --- |
| **A vs blank** | -3.40 | 1.1×10^-3^ | 6.15 |
| **B vs blank** | -3.73 | 5.0×10^-4^ | 5.38 |
| **C vs blank** | -3.19 | 1.9×10^-3^ | 5.90 |
| **D vs blank** | -3.84 | 4.0×10^-4^ | 5.13 |

**Supplementary Table 5. Non-parametric statistic tests carried out on chain spectra (Kolmogorov-Smirnov and Wilcoxon). The letters indicate TURBOGEN cylinders: A and C turbulent conditions; B and D still conditions. Internal controls indicate comparisons turbulent vs turbulent replicates (A-C) and still vs still replicates (B-D). TX (0≥X≥12) indicates time points. h indicates the test answer: 0 = null hypothesis accepted; 1 = null hypothesis rejected. The p-value associated to the test outcome is presented. Accuracy set at 99.9% (α = 10^-3^). The last rows (Total turbulent vs still) indicate run in pairwise among turbulent vs still samples merging results from the two replicates.**

| **Internal controls (turbulent vs turbulent and still vs still)** | | | | | | | | |
| --- | --- | --- | --- | --- | --- | --- | --- | --- |
|  | **Kolmogorov-Smirnov** | | | | **Wilcoxon** | | | |
|  | **A vs C** | | **B vs D** | | **A vs C** | | **B vs D** | |
|  | **h** | **p-value** | **h** | **p-value** | **h** | **p-value** | **h** | **p-value** |
| **T0** | 0 | 0.64 | 0 | 0.99 | 0 | 0.88 | 0 | 0.79 |
| **T1** | 0 | 9.24·10^-3^ | 0 | 0.99 | 0 | 0.01 | 0 | 0.74 |
| **T2** | 0 | 0.54 | 0 | 0.06 | 0 | 0.09 | 0 | 8.03·10^-3^ |
| **T3** | 1 | 7.24·10^-23^ | 0 | 0.06 | 1 | 5.12·10^-26^ | 0 | 8.26·10^-3^ |
| **T4** | 0 | 0.72 | 0 | 0.99 | 0 | 0.27 | 0 | 0.77 |
| **T5** | 0 | 6.86·10^-3^ | 0 | 0.63 | 0 | 2.28·10^-3^ | 0 | 0.11 |
| **T6** | 0 | 0.06 | 0 | 0.10 | 0 | 0.02 | 0 | 0.39 |
| **T7** | 0 | 0.37 | 1 | 5.46·10^-10^ | 0 | 0.16 | 1 | 1.69·10^-12^ |
| **T8** | 0 | 0.99 | 0 | 0.01 | 0 | 0.90 | 0 | 1.05·10-3 |
| **T9** | 0 | 0.22 | 1 | 3.34·10^- 5^ | 0 | 0.12 | 1 | 1.43·10^-6^ |
| **T10** | 0 | 0.42 | 0 | 0.29 | 0 | 0.10 | 0 | 0.03 |
| **T11** | 0 | 0.74 | 1 | 5.02·10^-4^ | 0 | 0.22 | 0 | 1.37·10^-3^ |
| **T12** | 0 | 0.19 | 0 | 0.16 | 0 | 0.24 | 0 | 0.03 |
| **Experimental comparisons (turbulent vs still)** | | | | | | | | |
| **Kolmogorov-Smirnov** | | | | | | | | |
|  | **A vs B** | | **A vs D** | | **C vs B** | | **C vs D** | |
| **T0** | 0 | 0.02 | 0 | 0.01 | 0 | 0.47 | 0 | 0.24 |
| **T1** | 1 | 6.52·10^-11^ | 1 | 5.16·10^-14^ | 1 | 2.09·10^-17^ | 1 | 2.53·10^-19^ |
| **T2** | 1 | 1.67·10^-4^ | 0 | 0.18 | 1 | 2.98·10^-7^ | 0 | 4.76·10^-3^ |
| **T3** | 1 | 1.55·10^-5^ | 0 | 0.03 | 1 | 3.01·10^-40^ | 1 | 1.88·10^-28^ |
| **T4** | 1 | 4.04·10^-8^ | 1 | 3.67·10^-7^ | 1 | 1.21·10^-10^ | 1 | 1.36·10^-10^ |
| **T5** | 1 | 5.89·10^-35^ | 1 | 1.34·10^-29^ | 1 | 1.33·10^-21^ | 1 | 3.96·10^-17^ |
| **T6** | 1 | 7.35·10^-17^ | 1 | 7.51·10^-13^ | 1 | 1.27·10^-11^ | 1 | 7.87·10^-6^ |
| **T7** | 1 | 9.24·10^-4^ | 1 | 2.51·10^-4^ | 0 | 0.01 | 1 | 3.48·10^-8^ |
| **T8** | 0 | 0.14 | 1 | 3.73·10^-5^ | 0 | 0.70 | 1 | 1.48·10^-5^ |
| **T9** | 0 | 0.11 | 1 | 6.00·10^-7^ | 0 | 0.12 | 1 | 1.11·10^-10^ |
| **T10** | 0 | 0.08 | 0 | 2.14·10^-3^ | 0 | 0.04 | 1 | 4.74·10^-5^ |
| **T11** | 0 | 0.03 | 1 | 1.74·10^-9^ | 0 | 1.14·10^-3^ | 1 | 1.39·10^-9^ |
| **T12** | 1 | 1.27·10^-10^ | 1 | 1.90·10^-5^ | 1 | 2.43·10^-10^ | 1 | 1.86·10^-5^ |
| **Wilcoxon** | | | | | | | | |
|  | **A vs B** | | **A vs D** | | **C vs B** | | **C vs D** | |
| **T0** | 0 | 0.05 | 0 | 0.10 | 0 | 0.05 | 0 | 0.10 |
| **T1** | 1 | 6.97·10^-16^ | 1 | 1.48·10^-17^ | 1 | 3.79·10^-23^ | 1 | 2.12·10^-25^ |
| **T2** | 1 | 8.33·10^-6^ | 0 | 0.07 | 1 | 1.64·10^-9^ | 1 | 5.38·10^-4^ |
| **T3** | 1 | 1.13·10^-8^ | 0 | 2.34·10^-3^ | 1 | 2.30·10^-44^ | 1 | 2.12·10^-35^ |
| **T4** | 1 | 5.10·10^-12^ | 1 | 2.46·10^-11^ | 1 | 5.77·10^-16^ | 1 | 3.30·10^-15^ |
| **T5** | 1 | 1.17·10^-41^ | 1 | 7.63·10^-36^ | 1 | 2.81·10^-26^ | 1 | 3.92·10^-21^ |
| **T6** | 1 | 6.50·10^-21^ | 1 | 8.36·10^-15^ | 1 | 1.56·10^-12^ | 1 | 9.77·10^-9^ |
| **T7** | 1 | 9.03·10^-5^ | 1 | 7.88·10^-5^ | 0 | 4.89·10^-3^ | 1 | 6.42·10^-7^ |
| **T8** | 0 | 0.70 | 1 | 1.53·10^-4^ | 0 | 0.57 | 1 | 2.06·10^-4^ |
| **T9** | 0 | 0.64 | 1 | 2.43·10^-6^ | 0 | 0.41 | 1 | 3.80·10^-9^ |
| **T10** | 0 | 0.56 | 0 | 2.95·10^-3^ | 0 | 0.044 | 1 | 1.36·10^-5^ |
| **T11** | 0 | 0.05 | 1 | 2.06·10^-6^ | 0 | 3.02·10^-3^ | 1 | 3.87·10^-8^ |
| **T12** | 1 | 2.42·10^-8^ | 0 | 2.32·10^-3^ | 1 | 6.49·10^-10^ | 1 | 1.27·10^-4^ |
| **Total turbulent vs still** | | | | | | | | |
|  | **Kolmogorov-Smirnov** | | | | **Wilcoxon** | | | |
|  | **h** | **p-value** | | | **h** | **p-value** | | |
| **T0** | 0 | 0.41 | | | 0 | 0.12 | | |
| **T1** | 1 | 4.36·10^-25^ | | | 1 | 4.91·10^-35^ | | |
| **T2** | 1 | 5.65·10^-4^ | | | 1 | 2.59·10^-4^ | | |
| **T3** | 1 | 3.64·10^-21^ | | | 1 | 5.77·10^-31^ | | |
| **T4** | 1 | 4.58·10^-13^ | | | 1 | 1.62·10^-21^ | | |
| **T5** | 1 | 3.33·10^-46^ | | | 1 | 6.48·10^-56^ | | |
| **T6** | 1 | 8.91·10^-16^ | | | 1 | 4.69·10^-22^ | | |
| **T7** | 0 | 0.04 | | | 0 | 0.32 | | |
| **T8** | 1 | 2.61·10^-4^ | | | 0 | 8.90·10^-3^ | | |
| **T9** | 1 | 3.00·10^-5^ | | | 1 | 3.55·10^-4^ | | |
| **T10** | 1 | 1.12·10^-4^ | | | 1 | 5.00·10^-4^ | | |
| **T11** | 1 | 2.38·10^-5^ | | | 1 | 1.26·10^-4^ | | |
| **T12** | 1 | 2.13·10^-11^ | | | 1 | 2.05·10^-7^ | | |
